# Quantitative Assessment of Choroidal Thickness and Choroidal Vascular Features in Healthy Eyes Based on Image Binarization of EDI-OCT: A Single-Center Cross-Sectional Analysis in Chinese Population

**DOI:** 10.3390/jcm12051911

**Published:** 2023-02-28

**Authors:** Luping Wang, Wei Wang, Zhuohua Zhou, Hao Wang, Usha Chakravarthy, Tunde Peto, Giuseppe Casalino, Kang Wang, Shuang Li

**Affiliations:** 1 Department of Ophthalmology, Beijing Friendship Hospital, Capital Medical University, Beijing 100050, China; 2 Department of Clinical Epidemiology and Evidence-Based Medicine, Beijing Clinical Research Institute, Beijing Friendship Hospital, Capital Medical University, Beijing 100045, China; 3 Department of Ophthalmology, Centre for Public Health, Queen’s University of Belfast, Belfast BT12 6BA, UK; 4 Eye Clinic, Fondazione IRCCS Cà Granda, Ospedale Maggiore Policlinico, University of Milan, 20122 Milan, Italy

**Keywords:** choroidal vascularity index, enhanced depth imaging optical coherence tomography, subfoveal choroidal thickness, luminal area

## Abstract

Purpose: To quantify the structural changes in choroidal vessels and to observe choroid microstructural changes in different age and sex groups in a healthy Chinese population. Methods: Enhanced depth imaging optical coherence tomography (EDI-OCT) was employed to analyze the luminal area, stromal area, total choroidal area, subfoveal choroidal thickness (SFCT), choroidal vascularity index (CVI), large choroidal vessel layer (LCVL), choriocapillaris–medium choroidal vessel layer, and LCVL/SFCT of the choroid in the subfoveal macular area within 1500 μm of the macula. We analyzed the age- and sex-related changes in the subfoveal choroidal structure. Results: A total of 1566 eyes from 1566 healthy individuals were included. The mean age of the participants was 43.62 ± 23.29 years, the mean SFCT of healthy individuals was 269.30 ± 66.43 μm, LCVL/SFCT percentage was 77.21 ± 5.84%, and the mean macular CVI was 68.39 ± 3.15%. CVI was maximum in the 0–10 years group, decreasing with age, and the lowest values occurred in the >80 years group; LCVL/SFCT was the lowest in the 0–10 years group, increasing with age and reaching a maximum in the >80 years group. CVI showed a significant negative correlation with age, and LCVL/SFCT showed a significant positive correlation with age. There was no statistically significant difference between males and females. Interrater and intrarater reliability was less variable with CVI than with SFCT. Conclusions: The choroidal vascular area and CVI decreased with age in the healthy Chinese population, of which the age-related decrease in vascular components maybe dominated by the decrease in choriocapillaris and medium choroidal vessels. Sex had no effect on CVI. The CVI of healthy populations showed better consistency and reproducibility when compared with SFCT.

## 1. Introduction

The choroid is the most vascularized structure in the eye, and choroidal vascular structures are correlated with the pathogenesis of various diseases, including age-related macular degeneration, polypoidal choroidal vasculopathy, central serous chorioretinopathy, and myopic macular degeneration [1,2,3,4,5]. Owing to the anatomical features of the choroid, structural analysis of the choroidal vasculature is challenging and there is a lack of qualitative and quantitative indicators. With the advent of enhanced depth imaging optical coherence tomography (EDI-OCT) technology, which is a modification of standard spectral-domain OCT (SD-OCT) and provides improved signal penetration, the study of the choroidal structure has advanced considerably [6,7,8,9]. Choroidal thickness (CT) is one of the most widely examined choroidal parameters, and has been found to be affected by different variables, such as axial length, refractive error, intraocular pressure, and systolic blood pressure, but does not provide us with information about structural changes in the choroid [10,11]. 

The application of EDI-OCT image binarization has become a research hotspot in recent years and has made it possible to explore the choroidal structure and its changes in different disease models. Choroidal vascularity index (CVI) is a novel parameter that is calculated from EDI-OCT scans via image binarization. It is defined as the ratio of vascular luminal area (LA) to total choroidal area (TCA), which is presented as a percentage. Several small-sample studies in healthy populations showed that CVI is less affected by physiological variables and has been demonstrated to be a reliable tool, which can quantitatively reflect structural changes in the choroidal vasculature [12,13,14]. CVI combined with subfoveal choroidal thickness (SFCT), LA, and stromal area (SA) can analyze choroidal morphological changes and choroidal blood perfusion.

In this study, we assessed the macular choroidal structure in a large sample of healthy Chinese individuals, and analyzed the changes in CVI and the thickness of each choroidal vascular layer with age and sex. We further examined the stability and reproducibility of CVI and SFCT measurements in this healthy population, and performed univariate and multivariate regression analyses.

## 2. Methods

### 2.1. Population

This cross-sectional study was conducted in the Department of Ophthalmology, Beijing Friendship Hospital, from December 2018 to December 2019. Individuals were enrolled according to the inclusion and exclusion criteria. Inclusion criteria: (1) best corrected visual acuity of ≥0.6 and (2) spherical power between +3D and −3D. Exclusion criteria: (1) patients who cannot undergo routine mydriasis; (2) active intraocular inflammation and/or infection, history of any type of intraocular surgery (except cataract surgery); (3) those with retinal and choroidal diseases (except retinal atherosclerosis); (4) those with significant refractive interstitial opacity or abnormalities that affect OCT measurements, such as significant corneal opacity, cataract, vitreous opacity, vitreous hemorrhage, or silicone oil inside the vitreous cavity; (5) those with hypertension, hyperlipidemia, diabetes, heart disease, cerebrovascular disease, pulmonary hypertension, renal disease, and peripheral and central vasculopathy; (6) current smoking and alcohol consumption; (7) those with acute or chronic infectious diseases, infectious inflammatory diseases, and malignancies; and (8) patients who are unable to cooperate in the examination or those in whom clear results cannot be obtained or whose test results cannot be analyzed. This study was approved by the Bioethics Committee of Beijing Friendship Hospital, Capital Medical University (2018-P2-205-01), and conducted in accordance with the tenets of the Declaration of Helsinki.

### 2.2. Data Collection

The data included demographic information (age, sex, occupation, marital status, education, and workload), health status (hypertension, diabetes mellitus, coronary atherosclerotic heart disease, hyperlipidemia, cerebrovascular disease, malignancy, peripheral vascular disease, etc.), and daily habits (alcohol consumption and smoking). Ocular data were collected from all enrolled patients, including best-corrected visual acuity, noncontact IOP measurements, slit lamp examination, and color fundus photography. Patients underwent high-definition EDI-OCT scans in both eyes using Spectralis OCT (Heidelberg Engineering, Heidelberg, Germany), and raster scans were performed to cover a 20 × 20° (6 × 6 mm) area. OCT images were acquired from 9:00 a.m. to 12:00 p.m. daily by the same technician (Wei Wang).

### 2.3. Quality Control of Images

Patients were enrolled by selecting EDI-OCT images of the left eye first and all data were collected by the same technician (Wei Wang). Three physicians in this study group (Shuang Li, Luping Wang, and Zhuohua Zhou) performed independent assessment of choroidal image clarity in both eyes. If two or more evaluators determined that the image clarity in the left eye was worse than that in the right eye, the patient was enrolled using the right eye image for analysis.

After the EDI-OCT images were acquired, three physicians from this study group performed independent evaluations. The images were considered acceptable and used for analysis when two or more investigators determined that the subfoveal choroid was clearly discernible and the choroidal and scleral boundaries were well demarcated. For images with an undefinable sclerochoroidal boundary, two physicians discussed the demarcation. If there was continuing discrepancy between them, the image was given to a third physician for arbitration and if a decision could not be reached, the image was excluded. Subsequent to this process, 27 images were eliminated, leaving 1566 images for consideration.

### 2.4. Range of the Subfoveal Choroidal Area

**Figure 1:**
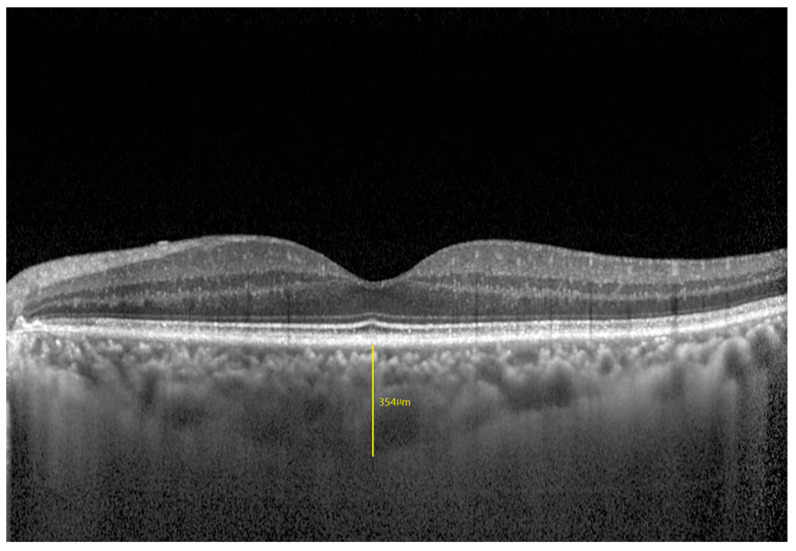
Subfoveal choroidal thickness measurement. The built-in software caliper tool was used to manually measure the vertical distance from the outer surface of the subfoveal retinal pigment epithelium (the highly reflective light band outside of Bruch’s membrane) to the sclerochoroidal junction.

SFCT: defined as the vertical distance from the outer surface of the subfoveal retinal pigment epithelium to the sclerochoroidal junction. SFCT was measured using the built-in software caliper tool (Figure 1).

### 2.5. Thickness Measurement of Each Choroidal Vascular Layer

**Figure 2:**
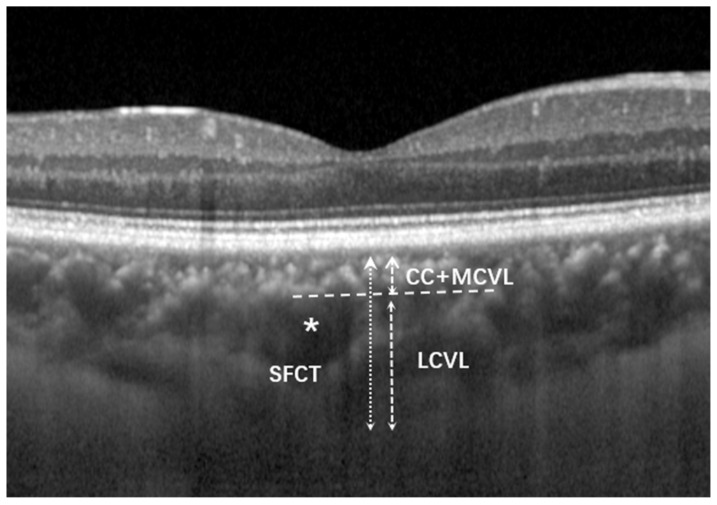
Thickness measurement of each vascular layer of the choroid in the healthy population. SFCT was measured at the fovea using the caliper of the OCT device. The asterisk (*) represents the large choroidal vessels (>100 μm in diameter) that are labeled closest to the fovea. A horizontal line was plotted along the inner edge of the large choroidal vessels to intersect the line measuring the SFCT. The thickness of the LCVL was measured from the inner edge of the sclerochoroidal junction to the innermost point of the selected large choroidal vessel. The choriocapillaris–medium choroidal vessel layer (CC+MCVL) thickness, which is the distance from the outer edge of the highly reflective retinal pigment epithelium to the dashed line, measured by subtracting the LCVL thickness from SFCT.

Vascular thickness analysis of each layer of the choroid was performed manually according to Branchini’s method [15]. The large choroidal vessels closest to the fovea were first selected. Large choroidal vessels were defined as those with a diameter of ≥100 μm. A horizontal line was drawn along the inner edge of the large choroidal vessels, and this line intersected the line used to measure the SFCT. Measurements were made from the sclerochoroidal junction to the point where this horizontal line intersected the SFCT measurement line. This length was taken as the thickness of the choroidal large vessel layer. The length of this intersection point to the Bruch’s membrane is equivalent to the thickness of the choriocapillaris–medium choroidal vessel layer (CC+MCVL), which was obtained by subtracting the large choroidal vascular layer (LCVL) thickness from the SFCT. We also calculated the ratio of LCVL to SFCT (Figure 2).

### 2.6. Image Binarization and CVI Measurement

**Figure 3:**
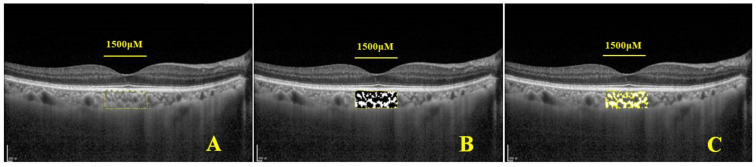
Choroidal image binarization and CVI measurement procedure: (**A**) Using the polygon selection tool in Image J, the choroidal region of interest (ROI) area was marked with Bruch’s membrane as the upper boundary and the sclerochoroidal interface as the lower boundary. The fovea was the center, and both nasal and temporal distances were 750 μm. The total choroidal area (TCA) of the subfovea was calculated. (**B**) Eight-bit conversion of the image was performed, and Niblack Auto Local Threshold tool was used to binarize the selected area. (**C**) After converting the image of selected area to RGB colors, the dark pixels were selected with the color threshold tool to calculate the area of luminal area (LA).

The public open-source software Image J (version 1.51) was used to process images (Figure 3). We masked patient information during image processing. The choroidal area centered on the fovea, and nasal and temporal distances of 750 μm were marked. The total choroidal area (TCA) was calculated. We performed 8-bit conversion of the image, and adopted Niblack Auto Local Threshold tool to binarize the selected area. In the binarized image, dark pixels indicated the vascular lumen and white pixels indicated the stromal region. After converting the image to RGB (red, green, and blue) colors, the dark pixels were selected using the color threshold tool, and the LA was calculated. SA was obtained by subtracting LA from TCA. The ratio of LA to TCA constituted CVI. Image segmentation was performed by one of the authors (Shuang Li) (Figure 3).

### 2.7. Interrater and Intrarater Agreement

CVI and SFCT were calculated by two examiners (Shuang Li and Zhuohua Zhou) to determine interrater agreement. The CVI and SFCT of the study cohort were calculated by one examiner (Shuang Li) after an interval of 1 week to compute intra-rater reliability. The interrater and intrarater reliability for the measurement of images was measured by the absolute agreement model of the intraclass correlation coefficient (ICC). ICCs of 0.81–1.00 indicate good agreement. ICCs < 0.3 indicate weak or poor agreement.

## 3. Data Analysis

The data were analyzed and processed using SPSS 24.0 software. The categorical data were analyzed using the χ^2^ test. Independent samples *t*-test and one-way analysis of variance test for normal distributions and Mann–Whitney U test and Kruskal–Wallis test for non-normal distributions were used to compare other parameters between the groups. Univariate and multivariate linear regression analyses were performed to determine the associations among the choroidal parameters, demographic profile, and various ocular factors. The coefficient of variation (CV) was calculated to compare the stability among multiple choroidal parameters. CV = (SD/Mean) × 100%. The difference was statistically significant when *p*-values were <0.05.

## 4. Results

### 4.1. Demographic Profile, Ocular and Choroidal Parameters of the Study Participants

In this study, 1566 patients were included and 27 were excluded, with a total of 1566 eyes, of which 768 eyes (49%) were in males and 798 eyes (51%) were in females. The mean age was 43.62 ± 23.29 years, ranging from 4 to 94 years. The body mass index (BMI), systolic blood pressure, diastolic blood pressure, fasting blood glucose, and ocular and choroidal parameters are summarized in Table 1. The mean age of men was 46.58 ± 25.81 years, and 46.80 ± 25.25 years for women, with no significant difference between the two groups (*p* = 0.886). There were no statistically significant differences in age, systolic blood pressure, diastolic blood pressure, fasting blood glucose, ocular characteristics (ocular axis, IOP), and choroidal parameters (TCA, SA, LA, CVI, SFCT, CC+MCVL thickness, LCVL thickness, and LCVL/SFCT) in males compared with those in females (Table 1). 

### 4.2. Aging and Choroidal Parameter Changes

Patients were grouped based on age, with every 10 years constituting one group. There were nine groups in total (0–10 years, 11–20 years, 21–30 years, 31–40 years, 41–50 years, 51–60 years, 61–70 years, 71–80 years, and >80 years groups). TCA, SA, LA, CVI, SFCT, CC+MCVL thickness, LCVL thickness, and LCVL/SFCT were compared across all nine age groups, and there were statistical differences (*p* < 0.05) among groups (Table 2 and Table 3).

**Figure 4:**
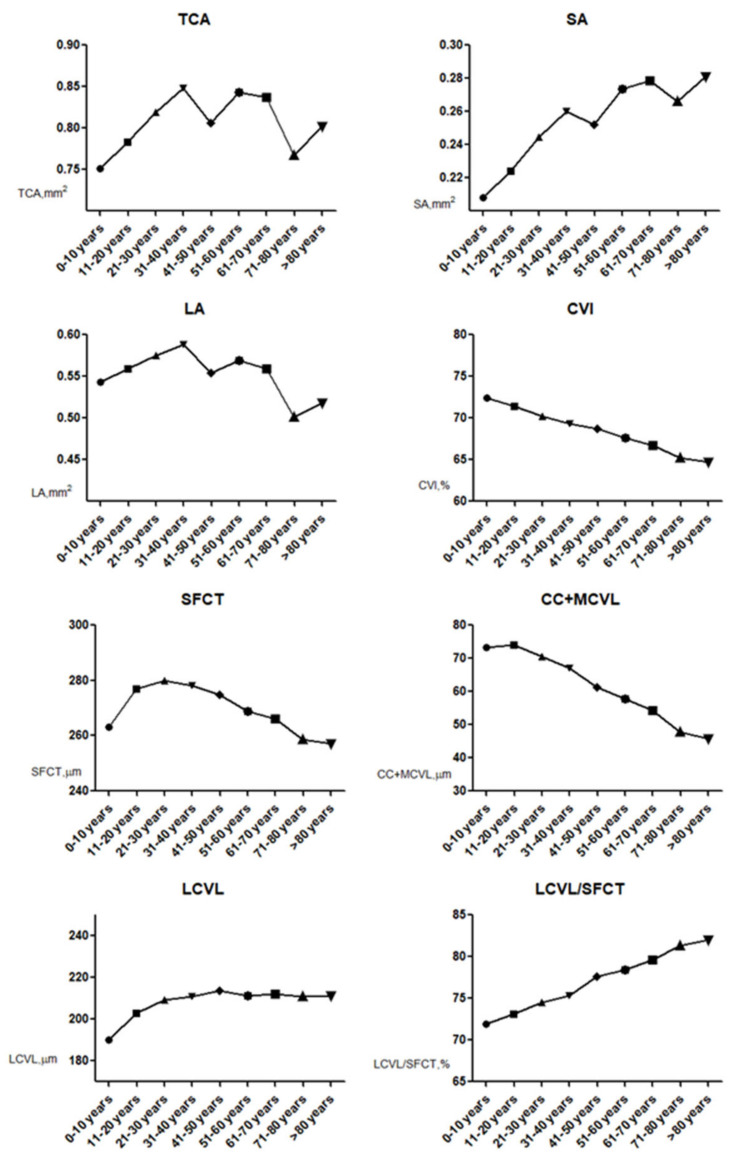
Age group curves for each choroidal parameter. CVI decreases gradually with age, and LCVL/SFCT increases gradually with age.

Figure 4 shows that the CVI was highest in the 0–10 years group (72.42 ± 2.10%) and gradually decreased with age, then reached the lowest value in the >80 years group (64.68 ± 3.12%). Similarly, SFCT and CC+MCVL thickness peaked in the 11–20 years (280.59 ± 44.23 μm, 75.37 ± 18.69 μm, respectively) and then decreased gradually with age, reaching a minimum in the >80 years group (257.24 ± 63.59 μm, 45.89 ± 14.05 μm, respectively). TCA and SA were least in the 0–10 years group (0.75 ± 0.17 mm^2^, 0.21 ± 0. 06 mm^2^, respectively). LA was lowest in the 71–80 years group (0.50 ± 0. 13 mm^2^) and >80 years age group (0.52 ± 0.15 mm^2^). LCVL/SFCT was the lowest in the 0–10 years (71.90 ± 6.78%), then gradually increased with age and reached the maximum in the >80 years age group (81.98 ± 4.34%).

### 4.3. Factors Influencing CVI and LCVL/SFCT Measurements

The univariate regression model revealed that age, BMI, ocular axis, LA, SA, LCVL thickness, CC+MCVL thickness, and LCVL/SFCT ratio were significantly correlated with CVI. However, only age, LA, LCVL thickness, and LCVL/SFCT ratio were correlated with CVI in the multiple regression model. Regression analysis showed that CVI decreased with increasing age (Table 4).

Age, ocular axis, TCA, LA, SA, CVI, SFCT, and LCVL thickness were significantly correlated with LCVL/SFCT on the univariate regression model. However, only age, TCA, LA, CVI, SFCT, and LCVL thickness were correlated with LCVL/SFCT ratio in the multiple regression model (Table 5).

### 4.4. Coefficient of Variation and Consistency Evaluation

The CV was 24.69% for TCA, 26.92% for LA, 25.45% for SA, 4.61% for CVI (LA/TCA), 24.67% for SFCT, 33.16% for CC+MCVL thickness, 26.42% for LCVL thickness, and 7.56% for the LCVL/SFCT ratio.

CVI and SFCT were calculated for normal healthy human eyes in the fovea at 1-week intervals by the same examiner, and intergroup consistency was observed using ICC. CVI relative consistency: ICC = 0.987 (95% confidence interval: 0.982–0.995); SFCT relative consistency: ICC = 0.964 (95% confidence interval: 0.946–0.975) (Table 6). 

## 5. Discussion

Recent advances in OCT imaging techniques, especially the EDI-OCT, have helped us better visualize the choroidal layers. Various OCT-related parameters have been developed to evaluate the choroid in healthy and disease states, and mostly evaluating the choroidal thickness and volume [15,16]. CVI is a noninvasive choroidal quantitative parameter, which is easily accessible [17]. The application of CVI has aroused great interest in studying the choroidal vascular structure [17,18]. The use of this quantitative parameter to assess the structural state of the choroid in healthy individuals at different ages can provide additional information for morphological and physiological structures of the choroid [19]. Furthermore, it can aid in observing age-related choroidal changes and help us to better characterize retinal/choroidal diseases, such as age-related macular degeneration, inflammatory chorioretinal disorders, pachychoroid disease spectrum, myopia, and inherited retinal disorders [20,21,22,23,24].

Sonoda et al. [15,24] first used image binarization to study LA and SA of the choroid in EDI-OCT images of healthy eyes. They reported a significant decrease in LA, SA, and LA/SA ratios with age, indicating a greater decrease in vascular LA than in SA. Ruiz-Medrano et al. [25] studied healthy individuals in a larger age range (3–85 years) and reported a significant decrease in LA and CVI with age while SA remained stable. However, the aforementioned study included only a limited sample size (136 individuals), the number of patients in each group was small, and data were missing for the younger children and the elderly population after stratifying by age [25].

Our study demonstrated a strong correlation between CVI and age in the macular region in this cohort of a healthy Chinese population, and the LA and CVI decreased with age. The SA was found to be smaller in the 0–10 and 11–20 age groups and gradually increased with age, reaching a maximum in the 61–70 age group, and then relatively stabilized. Previous histological studies have shown that the volume of choroidal cells and interstitial components decreases with age [26,27]. Several studies on choroidal immunohistochemistry reported a decrease in CT with age, accompanied by a significant decrease in collagen fibronectin, and cellular components in the choroid [26,27,28]. In addition, it is known that vascular tone and the amount of endothelial nitric oxide synthase (eNOS) decrease with age, thereby leading to a decrease in circulating blood volume [29,30]. All of these observations support the results of the present study. 

In our study population, both CVI and subfoveal LCVL/SFCT had less variability than that observed in SFCT. Moreover, previous studies showed that CVI and subfoveal LCVL/SFCT are less affected by physiological factors other than age [31]. These observations suggest that CVI and subfoveal LCVL/SFCT are relatively stable markers for studying choroidal changes. The CV of CVI was smaller compared with LCVL/SFCT in the fovea, indicating that CVI is a better and relatively more stable marker for monitoring the choroid and provides more information than a simple SFCT measurement.

The univariate analysis of ocular and systemic factors correlated with CVI showed significant correlations with age, BMI, ocular axis, LA, SA, LCVL thickness, CC+MCVL thickness, and LCVL/SFCT ratio. However, age, LA, LCVL thickness, and LCVL/SFCT ratio were factors correlated with CVI in the multiple regression model of this study. Of these, there was a strong correlation with age and a weak correlation with all other factors. Of note in the group of 0–10 years, the ocular axis is shorter, and the CVI is significantly higher. This may be due to the children at this stage having hyperopia and thicker choroid. In groups older than 10 years, natural growth and developmental processes of the eye, such as axial elongation and choroidal thinning, might play a role in reductions in CVI. We also observed that LCVL/SFCT showed a statistically significant correlation with age and CVI. Hence, understanding the age-related changes in choroidal structure in healthy eyes is important for the clinical application of CVI. This can better explain the differences in choroidal structure in patients of different ages and help explore the pathogenesis of various diseases, especially in ocular diseases with a strong correlation to age, such as age-related macular degeneration and adolescent myopia.

Furthermore, we observed a significant correlation between BMI and CVI in the univariate analysis. Since the current study included only healthy people with a mean BMI of 23.75 ± 5.72, we intend to study the high BMI population in future research, and analyze the changes in the choroidal vascular structure in the obese population.

Agrawal et al. reported that CVI was less variable than SFCT, and SFCT was affected by more factors than CVI [12]. In that population-based study, they included 345 healthy eyes. Except for CVI and SFCT, in the present study, we also measured LCVL, CC+MCVL, and LCVL/SFCT, expecting to find the correlation between the thickness of these two parts of the vascular layer and age. LCVL thickness was the smallest in the age group of 0–10 years, then gradually increased and peaked in the age group of 41–50 years, after which it slightly changed with age. CC+MCVL thickness was the maximum in the age group of 11–20 years, then gradually decreased with age and reached the minimum after 80 years. LCVL/SFCT was the smallest in the group of 0–10 years, then increased gradually with age and reached the maximum in the group of >80 years. LCVL thickness changed very little with age >20 years, but CC+MCVL decreased significantly with age. We inferred that some of the choriocapillaris may occlude and detach with age, which may also be correlated with the occurrence of age-related macular degeneration. The CV of LCVL/SFCT was 7.56%. It is also a relatively stable biological parameter for monitoring choroidal vascular structures, an understanding of its age-related changes in normal eyes could enable further exploration of the pathogenesis of various age-related fundus diseases. We further observed the measurement consistency of CVI and SFCT, and the repeatability of both parameters was within the confidence range. Compared to SFCT, the ICC values of CVI were significantly higher and exhibited better measurement consistency.

The strengths of our study are (1) It is a large-sample observation with a single common ethnicity, and is less likely to be confounded by ethnic heterogeneity. (2) Our study applied standardized clinical examination protocols and image processing procedures. (3) We confirmed that CVI and LCVL/SFCT had less variability than that observed in SFCT. However, this study has several limitations: (1) Binarization of the images was only conducted in the single eye of each study subject. (2) Although OCT images were binarized at standardized protocols, there was a possibility of over or underestimation of LA and SA. (3) Our study mainly focused on the subfoveal region, the extramacular choroidal vascular features in individuals have remained largely unexplored.

In conclusion, in this study based on a large sample of subjects, we found that CVI of healthy populations showed better consistency and reproducibility when compared with SFCT. In addition, we demonstrated that the choroidal vascular area and CVI significantly decrease with age. Age-related decrease in vascular components maybe dominated by a decrease in CC+MCVL. Our findings provide new insights that may be helpful in future studies on the pathophysiology of the human choroid. Larger datasets in different disease states are needed to further validate the value of these markers for application in clinical practice.

## Figures and Tables

**Table 1:** Demographic profile and comparison of sex subgroups.

Sample Size	Total	Male	Female	*p*-Value
	n = 1566	n = 768	n = 798	
Age, years	43.62 ± 23.29	46.58 ± 25.81	46.80 ± 25.25	0.886
BMI, kg/m^2^	23.75 ± 5.72	23.49 ± 5.60	23.87 ± 5.77	0.186
Systolic blood pressure, mmHg	105.21 ± 13.63	105.85 ± 12.98	104.92 ± 13.71	0.169
Diastolic blood pressure, mmHg	67.27 ± 10.85	68.07 ± 11.02	67.13 ± 10.42	0.083
Fasting blood glucose, mmol/L	4.38 ± 1.35	4.43 ± 0.87	4.37 ± 1.52	0.340
Ocular axis, mm	22.91 ± 1.02	22.85 ± 2.13	22.92 ± 1.84	0.486
IOP, mmHg	13.69 ± 2.37	13.59 ± 2.43	13.71 ± 1.95	0.280
TCA, mm^2^	0.81 ± 0.20	0.81 ± 0.20	0.82 ± 0.20	0.516
SA, mm^2^	0.25 ± 0.07	0.25 ± 0.07	0.26 ± 0.07	0.709
LA, mm^2^	0.56 ± 0.14	0.56 ± 0.14	0.56 ± 0.14	0.449
CVI (LA/TCA), %	68.68 ± 2.97	68.60 ± 2.97	68.76 ± 2.97	0.306
SFCT, μm	271.64 ± 67.35	271.50 ± 70.60	271.78 ± 64.12	0.934
CC+MCVL thickness, μm	62.60 ± 20.62	61.97 ± 20.75	63.21 ± 20.49	0.234
LCVL thickness, μm	209.04 ± 55.74	209.53 ± 58.07	208.57 ± 53.44	0.733
LCVL/SFCT, %	76.81 ± 5.91	77.03 ± 5.68	76.6 ± 6.12	0.145

IOP: intraocular pressure; BMI: body mass index; TCA: total choroidal area; LA: luminal area; SA: stromal area; CVI: choroidal vascularity index; SFCT: subcentral fovea choroidal thickness; LCVL: large choroidal vessel layer; CC+MCVL: choriocapillaris–medium choroidal vessel layer.

**Table 2:** Comparison of TCA, SA, LA, and CVI in various age groups.

	n	TCA, mm^2^	SA, mm^2^	LA, mm^2^	CVI (LA/TCA), %
0–10 years old group	115	0.75 ± 0.17	0.21 ± 0.06	0.54 ± 0.12	72.42 ± 2.10
11–20 years old group	163	0.80 ± 0.17	0.23 ± 0.050	0.56 ± 0.12	71.41 ± 1.58
21–30 years old group	255	0.82 ± 0.21	0.24 ± 0.07	0.57 ± 0.15	70.29 ± 1.84
31–40 years old group	228	0.85 ± 0.18	0.26 ± 0.05	0.59 ± 0.13	69.29 ± 1.87
41–50 years old group	212	0.81 ± 0.19	0.25 ± 0.06	0.55 ± 0.13	68.73 ± 1.66
51–60 years old group	205	0.84 ± 0.22	0.27 ± 0.07	0.57 ± 0.15	67.59 ± 1.87
61–70 years old group	133	0.84 ± 0.20	0.28 ± 0.07	0.56 ± 0.13	66.71 ± 1.63
71–80 years old group	129	0.77 ± 0.20	0.27 ± 0.07	0.50 ± 0.13	65.21 ± 2.03
>80 years old group	126	0.80 ± 0.24	0.28 ± 0.09	0.52 ± 0.15	64.68 ± 3.12
*p*-value		<0.001 *	<0.001 *	<0.001 *	<0.001 *

* Indicates that the difference is statistically significant.

**Table 3:** Comparison of SFCT, CC+MCVL thickness, LCVL thickness, and LCVL/SFCT in various age groups.

	n	SFCT, μm	CC+MCVL Thickness, μm	LCVL Thickness, μm	LCVL/SFCT, %
0–10 years old group	115	263.07 ± 50.41	73.20 ± 19.82	189.87 ± 42.66	71.90 ± 6.78
11–20 years old group	163	280.59 ± 44.23	75.37 ± 18.69	205.23 ± 38.98	73.02 ± 5.97
21–30 years old group	255	280.21 ± 76.75	70.65 ± 24.82	209.56 ± 64.46	74.50 ± 7.43
31–40 years old group	228	277.79 ± 91.75	67.21 ± 22.90	210.58 ± 74.69	75.35 ± 5.74
41–50 years old group	212	274.96 ± 59.10	61.32 ± 13.56	213.65 ± 47.54	77.61 ± 2.39
51–60 years old group	205	268.89 ± 58.35	57.68 ± 13.07	211.20 ± 47.36	78.43 ± 2.81
61–70 years old group	133	266.26 ± 56.78	54.23 ± 12.22	212.04 ± 45.94	79.60 ± 1.89
71–80 years old group	129	258.67 ± 69.53	47.84 ± 15.78	210.83 ± 58.20	81.35 ± 3.69
>80 years old group	126	257.24 ± 63.59	45.89 ± 14.05	211.35 ± 55.69	81.98 ± 4.34
*p*-value		0.003 *	<0.001 *	0.029 *	<0.001 *

* Indicates that the difference is statistically significant.

**Table 4:** Linear regression analysis of ocular and systemic factors correlated with CVI.

	Univariate			Multivariate		
	Unstandardized β	Standardized b	*p*-Value	Unstandardized β	Standardized b	*p*-Value
Age/10 years	0.951	−0.745	<0.001	−0.084	−0.065	<0.001
Sex	−0.003	−0.021	0.869	/	/	/
BMI	−0.005	−0.290	0.050	−0.003	−0.164	0.190
Diastolic blood pressure	−0.001	−0.032	0.735	/	/	/
Systolic blood pressure	−0.000	−0.016	0.893	/	/	/
Fasting blood glucose	−0.001	−0.036	0.643	/	/	/
Ocular axis	−0.003	−0.138	0.040	−0.001	−0.099	0.132
IOP	−0.001	−0.034	0.704	/	/	/
TCA mm^2^	−0.011	−0.009	0.756	/	/	/
SA, mm^2^	−0.029	0.403	<0.001	−0.013	−0.354	0.231
LA, mm^2^	0.017	0.312	<0.001	36.830	1.712	<0.001
SFCT, μm	0.009	−0.019	0.752	/	/	/
CC+MCVL thickness, μm	−0.003	0.462	0.011	−0.003	−0.338	0.160
LCVL thickness, μm	0.002	0.534	<0.001	0.003	−0.058	0.011
LCVL/SFCT	0.000	0.248	<0.001	−0.034	−0.068	0.012

CVI: choroidal vascular index; TCA: total macular choroidal area; SA: stromal area; LA: luminal area; SFCT: subcentral fovea choroidal thickness; LCVL: large choroidal vessel layer; CC+MCVL: choriocapillaris–medium choroidal vessel layer.

**Table 5:** Linear regression analysis of ocular and systemic factors correlated with LCVL/SFCT.

	Univariate			Multivariate		
	Unstandardized β	Standardized b	*p*-Value	Unstandardized β	Standardized b	*p*-Value
Age, /10 years	−1.302	0.513	<0.001	0.850	0.062	0.013
Sex	−0.003	−0.021	0.869	/	/	/
Ocular axis	−0.003	−0.138	0.03	−0.003	−0.093	0.193
IOP	−0.001	−0.034	0.704	/	/	/
BMI	−0.002	−0.790	0.562	/	/	/
Diastolic blood pressure	−0.002	−0.027	0.795	/	/	/
Systolic blood pressure	−0.001	−0.019	0.587	/	/	/
Fasting blood glucose	−0.001	−0.006	0.685	/	/	/
TCA mm^2^	−0.003	−0.027	<0.001	−0.002	−0.084	0.024
SA, mm^2^	−0.002	−0.585	<0.001	−0.001	−0.503	0.374
LA, mm^2^	0.001	0.297	0.034	0.000	−0.447	0.001
SFCT, μm	0.002	−0.019	<0.001	−0.002	−0.094	<0.001
CC+MCVL thickness, μm	−0.001	−0.02	0.538	/	/	/
LCVL thickness, μm	0.003	0.032	<0.001	0.003	−0.047	<0.001
CVI	0.004	0.302	<0.001	0.003	−0.364	0.001

TCA: total macular choroidal area; SA: stromal area; LA: luminal area; SFCT: subcentral fovea choroidal thickness; LCVL: large choroidal vessel layer; CC+MCVL: choriocapillaris–medium choroidal vessel layer; CVI: choroidal vascular index.

**Table 6:** Interrater and intrarater reliability assessment of CVI and SFCT measurements.

	Interrater Reliability		Intrarater Reliability	
	ICC	95% Confidence Interval	ICC	95% Confidence Interval
CVI	0.987	0.967–0.991	0.987	0.982–0.995
SFCT	0.962	0.937–0.982	0.964	0.946–0.975

CVI: choroidal vascular index; SFCT: subcentral fovea choroidal thickness; ICC: intraclass correlation coefficient.

## Data Availability

The data presented in this study are available on request from the corresponding author.
